# Immunotherapy and gene therapy as novel treatments for cancer

**DOI:** 10.25100/cm.v48i3.2997

**Published:** 2017-09-30

**Authors:** Martha Montserrat Rangel-Sosa, Estuardo Aguilar-Córdova, Augusto Rojas-Martínez

**Affiliations:** 1 Departamento de Bioquímica y Medicina Molecular, Facultad de Medicina, Universidad Autónoma de Nuevo León. Nuevo León, México.; 2 Advantagene, Auburndale, MA USA.; 3 Escuela de Medicina y Ciencias de la Salud. Tecnológico de Monterrey. Monterrey, México.

**Keywords:** cancer, immunotherapy, monoclonal antibody, regulatory T cells, gene therapy, cáncer, inmunoterapia, anticuerpo monoclonal, células T reguladoras, terapia génica

## Abstract

The immune system interacts closely with tumors during the disease development and progression to metastasis. The complex communication between the immune system and the tumor cells can prevent or promote tumor growth. New therapeutic approaches harnessing protective immunological mechanisms have recently shown very promising results. This is performed by blocking inhibitory signals or by activating immunological effector cells directly. Immune checkpoint blockade with monoclonal antibodies directed against the inhibitory immune receptors CTLA-4 and PD-1 has emerged as a successful treatment approach for patients with advanced melanoma. Ipilimumab is an anti-CTLA-4 antibody which demonstrated good results when administered to patients with melanoma. Gene therapy has also shown promising results in clinical trials. Particularly, *Herpes simplex* virus (HSV)-mediated delivery of the HSV thymidine kinase (TK) gene to tumor cells in combination with ganciclovir (GCV) may provide an effective suicide gene therapy for destruction of glioblastomas, prostate tumors and other neoplasias by recruiting tumor-infiltrating lymphocytes into the tumor. The development of new treatment strategies or combination of available innovative therapies to improve cell cytotoxic T lymphocytes trafficking into the tumor mass and the production of inhibitory molecules blocking tumor tissue immune-tolerance are crucial to improve the efficacy of cancer therapy.

## Resumen

El sistema inmune interactúa íntimamente con los tumores durante el proceso del desarrollo de la enfermedad y su progresión a metástasis. Esta compleja comunicación entre el sistema inmune y las células tumorales puede prevenir o promover el crecimiento del tumor. Los nuevos enfoques terapéuticos que aprovechan los mecanismos inmunológicos, ya sea por el bloqueo de señales inhibitorias o por la activación directa de células efectoras, han mostrado resultados prometedores. El bloqueo de puntos de control inmunológicos *immune-checkpoints* con anticuerpos monoclonales dirigidos contra receptores que normalmente inhiben el sistema inmune, como CTLA-4 o PD-1, ha resultado ser un tratamiento exitoso para pacientes con melanoma avanzado. El fármaco ipilimumab es un anticuerpo anti-CTLA-4 que ha demostrado buenos resultados terapéuticos en pacientes con melanoma. Por otro lado, la terapia génica también ha mostrado resultados prometedores en ensayos clínicos. En especial, la administración de la enzima timidina quinasa del virus *Herpes simplex* (HSV-TK) en combinación con el fármaco ganciclovir (GCV) ha mostrado ser una terapia suicida muy efectiva para la destrucción de diferentes neoplasias incluyendo glioblastomas y tumores prostáticos, por un mecanismo que involucra el reclutamiento de linfocitos infiltrantes de tumor. Es importante la búsqueda de nuevas estrategias o la combinación de terapias innovadoras, con el fin de involucrar tanto la atracción de linfocitos citotóxicos así como el empleo de moléculas que inhiban la inmunotolerancia del tejido tumoral para mejorar la eficiencia de los tratamientos contra el cáncer.

## Introduction

 Cancer progression is accompanied by a strong suppression of the immune system (IS), which interferes with effective antitumor response and diminishes tumor eradication ^1^. The immune-surveillance evasion occurs, in part, due to the fact that the tumor microenvironment inhibits T cell proliferation and attracts immune-suppressor cells ^2^. 

 A better knowledge of the interaction between the tumor and the IS has allowed the development of specific therapies designed to improve patient´s immune response. Tumor immunotherapy has two strategies: attack the tumor directly or activate the IS by the use of cell therapies, like stimulatory agonists or the immune-checkpoint blockade ^3^; The latter has demonstrated a potential antitumoral immune response, proving to be a promising therapy ^4^. Another option is the use of a different approach: gene therapy, which allows modifying tumor gene expression for therapeutic purposes. For example, tumor cell transduction with "suicide genes" is a largely investigated strategy of anti-neoplastic gene therapy ^5^. 

 This article reviews the use of immune-checkpoint blockade and suicide gene therapy as different alternatives for cancer therapy and analyzes the possible synergic effects that can be reach with the combination of this both therapies.

## 1. Cancer and immune system

The IS interacts intimately with the tumors during the process of disease development and its progression to metastasis (tumoral immunology) ^6^. It also respond to cancer by recognizing and eliminating the abnormal cells (immuno-surveillance) ^7^. However, some resistant cells can evade this control (immunoediting) ^8^ reducing their immunogenicity ^9^ and promoting malignant growth ^7^.

 Tumor cells change their surface markers recurrently.. For example, they express tumor-associated antigens (TAA) ^10^ or reduce the expression of the major histocompatibility complex (MHC) class I. This can lead to the activation of the innate immune response cells, such as natural killer (NK) cells ^10^. Macrophages and neutrophils may attack the tumor cells and stimulate the cytotoxic T lymphocytes (CTL), the antigen-presenting cell (APC) and the NK cells. In contrast, inflammatory cells produce growth factors and angiogenesis-stimulating growth factors promoting tumor growht ^11^.

In the adaptive response, the processed TAA are presented by the MHC class I and II molecules from APCs to the specific receptors of T CD8+ and CD4+ cells respectively for their activation ^10^ ^,^ ^12^. The CD8+ T lymphocytes are considered the main antitumor effector cells ^13^. Once activated, they mediate the lysis of tumor cells ^10^. Among the CD4+ T cells, the Th1 are responsible for cellular immunity: they secrete interleukine (IL)-2, TNFα and interpheron-γ (IFN-γ), promote the macrophage´s cytotoxic activity and induce the overexpression of MHC I and III in the APC. In contrast, the Th2 cells express IL-4, -5, -10 and -13, inducing clonal anergy, enhancing humoral immunity and regulating macrophage activity ^13^. On the other hand, the regulatory T (Treg) cells help to reduce inflammation by the production of TGF-β, IL-35 e IL-10 ^10^. The tumor cells can secrete chemokines as CCL22 which recruit Treg cells to supress the effector function of T cells and decreasing the immune response ^10^.

Tumors can also deregulate the IS by altering a complex balance between activating and inhibitory signals (checkpoints) in different pathways that regulate the function of T cells ^7^. 

## 2. Regulatory T cells

Treg cells are relevant to the maintenance of the immunological homeostasis: they preserve the tolerance to self-antigens, prevent the autoimmune diseases, modulate the development of an immune response and favor the escape of tumor cells from immune control ^14^ ^,^ ^15^. The best-characterized subpopulation expresses CD4, CD25 and Foxp3. Treg can suppress different cells such as CD4+ and CD8+ T lymphocytes, natural killer T cells, dendritic cells (DC), monocytes/macrophages, B lymphocytes and NK cells ^14^.

Because Treg suppresses the immune response against self-antigens ^8^ ^,^ ^16^, it is postulated that TAA may induce an increase in the number of intratumoral Treg cells in several neoplasms, including colorectal cancer (CRC), facilitating tumor immunotolerance ^8^ ^,^ ^17^. The accumulation of Treg in tumors is explained by several mechanisms, such as the conversion of CD4+ T cells to Treg in response to membrane-bound TGF-β, the recruitment of Tregs by chemokines as CCL17, CCL22 and CCL28 and tumor secretion of VEGF-A in response to hypoxia, which inhibits DC maturation. Immature DCs express TGF-β favoring the conversion of CD4 + T cells to Treg ^18^.

The most frequents TAA are own-antigens subexpressed in normal cells but highly expressed in tumor cells ^18^. One of the best known is the carcinoembryonic antigen (CEA) which is highly expressed in CRC ^18^; the CEA is recognized as a self-antigen by the Tregs ^19^, causing a poor immune response to tumor cells. In ovarian, breast, pancreatic, stomach and liver cancers, an increase in Treg lymphocytes in the tumor is associated with a worse prognosis ^17^. The use of these cells as targets may benefit the therapeutic strategies against cancer ^8^.

### 2.1 Action mechanisms of the Treg cells

**Figure 1:**
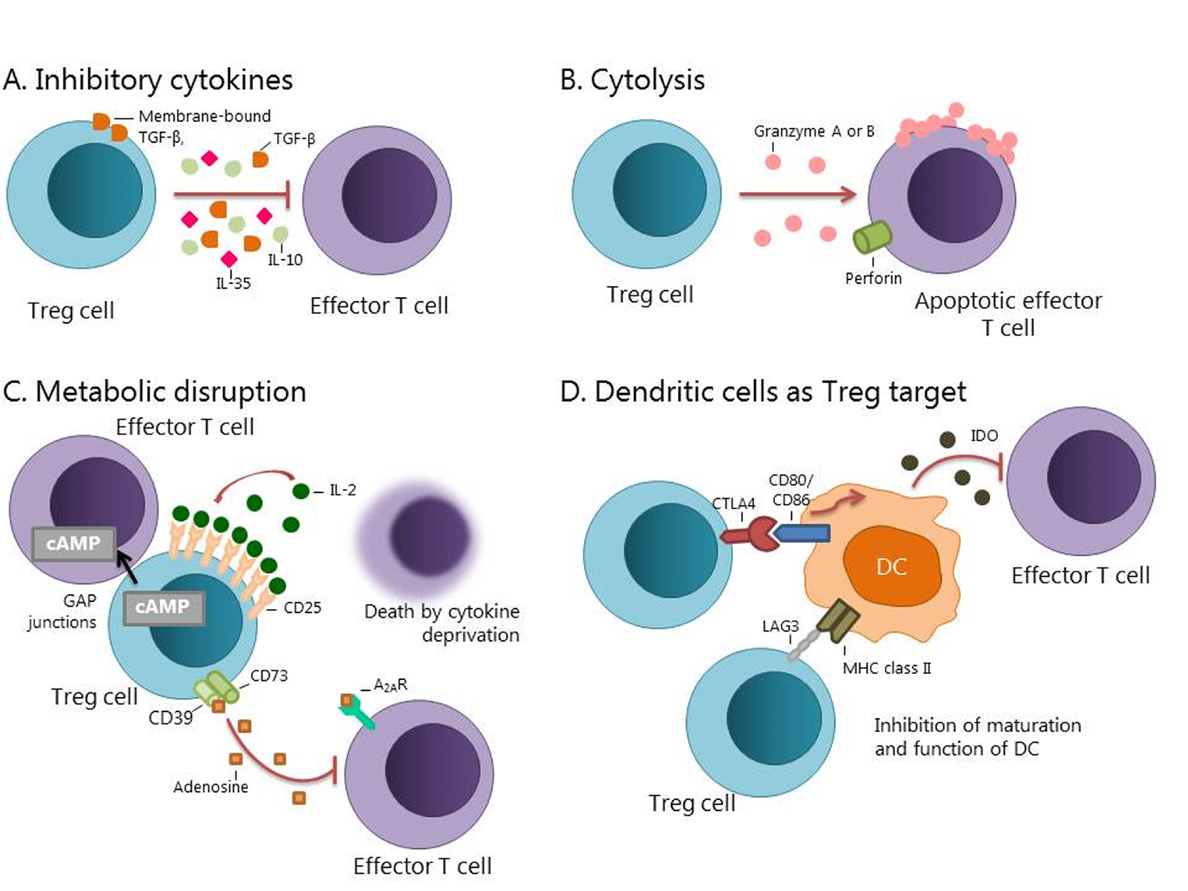
Action mechanism of Treg cells. A. Production of inhibitory cytokines such as IL-10, IL-35, and TGF-β. B. The inhibition by cytolysis includes dependent mechanism of granzyme A or granzyme B as well as perforin dependent mechanisms. C. A metabolic disruption can occur by Treg cells due an overproduction of CD25, capturing IL-2; inhibition by cAMP or immunosuppression through the adenosine 2A receptor. D. The function and maturation of the DC can be modulated by the LAG3, CTLA-4 or the enzyme IDO pathways (Modified by Vignali et al. 2008) ^20^.

The Treg lymphocytes have four main mechanisms of action to regulate the immune response Fig. 1. The first is suppression by inhibitory cytokines, which include IL-3, IL-10 and TGF-β ^18^^,^^20^ . The second is suppression by cytolysis. Tregs may induce cytolysis of B cells through the production of granzyme B. These cells may also exert a cytolytic effect to CD8+ T lymphocytes and NK cells by granzyme-B-dependent and perforin-dependent killing mechanisms, or by the aTRAIL-DR5^20^ ^-^ ^22^ pathway ^20^ ^-^ ^22^. The third mechanism is the suppression by metabolic alterations that affect the activity of molecules such as CD25 (IL-2 receptor), cyclic AMP (cAMP), CD39, CD73 and adenosine 2A receptor (A2AR) ^20^. The fourth mechanism consists on the suppression of the maturation and/or function of DC. This includes pathways such as the lymphocyte-activation gene-3 (LAG3) or the interaction between cytotoxic T-lymphocyte associated protein 4 (CTLA-4) and CD80/86, which induces the enzyme indoleamine 2,3-dioxygenase (IDO), an immunosuppressive molecule generated by DC ^10^ ^,^ ^20^. The blocking of these immunosuppressive mechanisms could increase the function of T cells and generate a more effective clinical response ^8^ ^,^ ^20^.

### 2.2 Regulation of immune-checkpoints by Treg cells

In the case of T cells, the amplitude and quality of the response after recognition of an antigen is regulated by a balance between costimulatory and inhibitory signals (immune-checkpoint) ^4^. In order to increase the effector function of tumor-infiltrating T cells, the immunosuppressive signals can be inhibited. There has been a greater clinical success with this strategy in the treatment of several types of cancer, such as melanoma and lung cancer ^6^. Some molecules that act as immune-checkpoints, such as CTLA-4 receptor and programmed cell death protein 1 (PD-1), are expressed on Treg lymphocytes and tumor-infiltrating effector T cells ^15^. 

### 2.3 Cancer immune-checkpoints 

As previously mentioned, the activation and inhibition of different receptors regulate the balance between immune response and immunotolerance ^23^ ^,^ ^24^, which is important for complete activation and effector function of T cells ^25^. The antibody therapy against negative immunological regulators has shown success in antineoplastic therapy ^23^ ^,^ ^25^, because it increases the potential of the antitumor immune response. 

It has been demonstrated that tumors use some immunological control pathways as a mechanism of immune resistance ^4^, e.g. increasing the expression of the checkpoint proteins decreases the function of T cells. Examples of immunological checkpoints are PD-1, CTLA-4, LAG-3, T cell immunoglobulin and mucin domain-containing protein 3 (TIM-3), among others ^26^.

Antitumor antibodies that block immune-checkpoints are directed against lymphocyte receptors or their ligands ^27^ Table 1. Two immune-checkpoints widely studied in the clinical context of cancer immunotherapy are CTLA-4 and PD-1. Both are inhibitory receptors that regulate the immune response ^27^. Although inhibition of control points seems to be successful in the treatment of some cancers, adverse events are associated, in particular autoimmune responses affecting organs such as the colon, skin, some endocrine glands, liver, etc ^26^.

**Table 1:** Development of pharmacological agents directed against immune-checkpoints signaling pathways ^36^ ^,^ ^80^.

Target	Biological function	Antibodie	Clinical situation
CTLA-4	Inhibitory receptor	Ipilimumab	Approved by the FDA for melanoma. Phase IV trials for melanoma and metastatic renal cell cancer. Phase III trials for stomach / esophagus cancer, small cell and non-small cell lung cancer, renal carcinoma, pleural mesothelioma, metastatic squamous cell carcinoma of the head and neck, prostate cancer, ocular melanoma.
		Tremelimumab	Tested in phase III trials for melanoma, head and neck cancer, small and non-small cell lung cancer, urothelial cancer.
PD-1	Inhibitory receptor	Nivolumab	FDA approved for melanoma, renal carcinoma, non-small cell lung cancer. Phase IV trials for advanced metastatic renal carcinoma and metastatic melanoma. Phase III trials for small cell and non-small cell lung cancer, stomach/esophagus cancer, melanoma, mesothelioma, hepatocellular carcinoma, multiple myeloma, urothelial cancer, gastric cancer.
		Pembrolizumab	FDA approved for melanoma, non-small cell lung cancer. Phase III trials for melanoma and small and non-small cell lung cancer.
		Pidilizumab	Phase I/II trials for lymphoma, multiple myeloma, pancreatic cancer.
PD-L1	Programmed death-ligand 1	BMS-936559	Phase I trials for melanoma.
		Atezolizumab	Phase III trials for small cell and non-small cell lung cancer, triple negative breast cancer, urinary tract cancer, renal cancer, ovarian cancer, colorectal cancer, melanoma.
LAG3	Inhibitory receptor	IMP321	Phase I / II trials for breast adenocarcinoma, renal carcinoma, melanoma, pancreatic neoplasms.
B7-H3	Inhibitory ligand	Enoblituzumab	Phase I trials for various types of cancer.

CTLA-4: Cytotoxic T-Lymphocyte Antigen 4;

LAG3: Lymphocyte-activation gene 3;

PD-1: Programmed cell death protein 1;

PD-L1: Programmed death-ligand 1.

### 2.4 CTLA-4

The CTLA-4 receptor is the first immune-checkpoint used as clinical target ^28^. CTLA-4 is a member of the immunoglobulin superfamily CD28:B7. It is normally expressed at low levels on the surface of effector T cells and Treg cells. Its function is to regulate the amplitude of the early stages activation of these kinds of cells ^28^. To activate a T cell, three signals are required: the antigen binding to the T cell receptor (TCR), the interaction of MHC (in human: human leukocyte antigen, HLA) with CD8 or CD4 T cell receptors, and the generation of a costimulatory signal generated by the binding of CD80(B7) to CD28 ^12^ ^,^ ^29^. Once this is completed, the CD28 pathway amplifies the TCR signaling to activate T cell proliferation. CD28 and CTLA-4 share the same ligands: CD80 (B7.1) and CD86 (B7.2), however these ligands bind to CTLA-4 with higher affinity ^4^ ^,^ ^30^, because of this, CTLA-4 counteracts the costimulatory activity of CD28 ^4^ ^,^ ^31^. 

CTLA-4 is crucial in T-cell activation. This is demonstrated by the lethal phenotype of the hyperactivated immune system in CTLA-4 knockout mice ^4^. Although CTLA-4 is expressed in activated effector CD8+ T cells, its most important physiological function is through different effects on CD4+ T cells: the activity decrease of helper T cells (Th1) and the enhancing of the immunosuppressive activity of Treg cells ^4^. 

**Figure 2:**
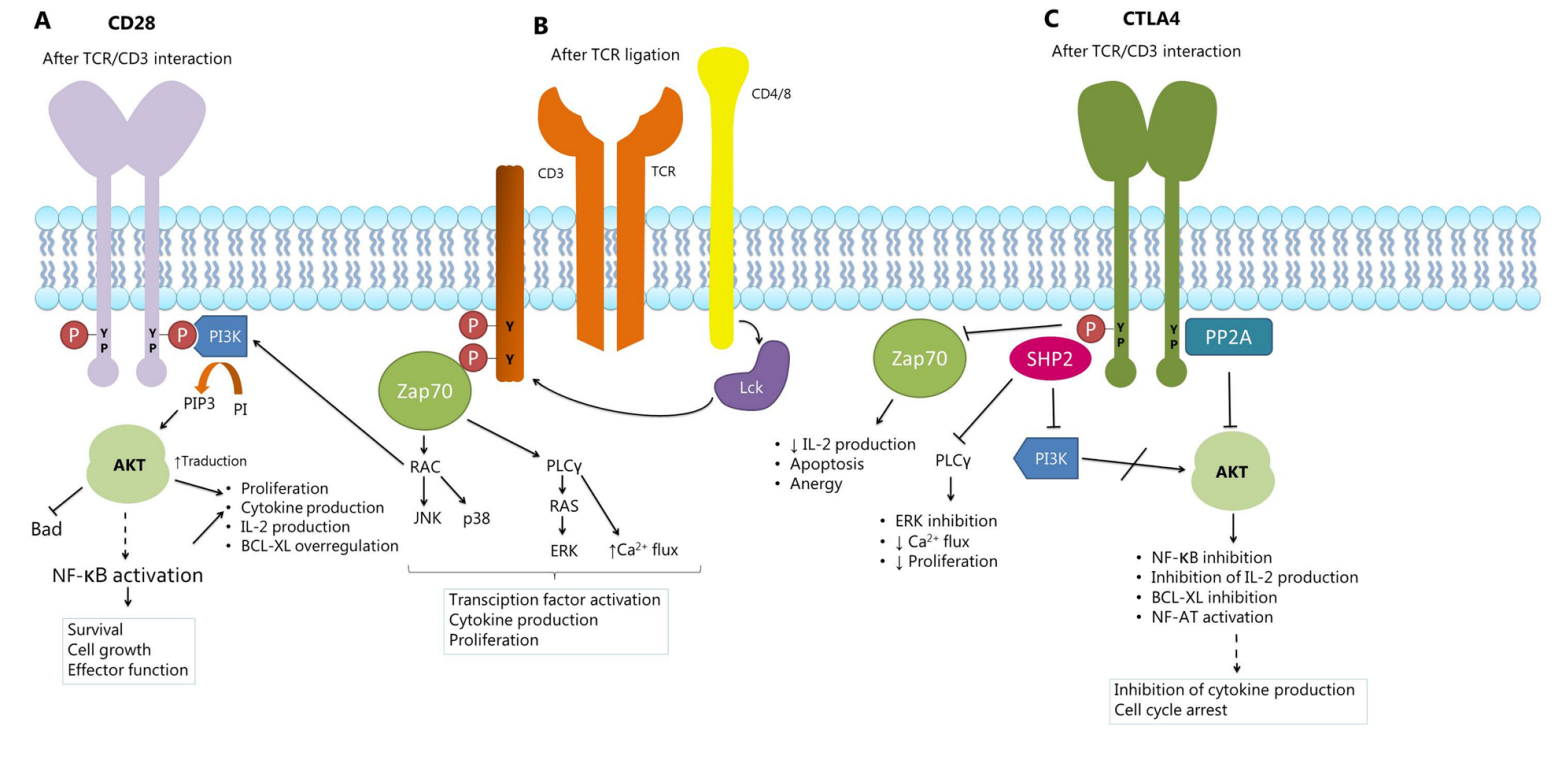
Signaling model of CD28 and CTLA-4. A. When T cell stimulation occurs, the intracellular tyrosine residues of CD28 are phosphorylated, and this attracts kinase 3 phosphatidylinositol (PI3K). The activation of PI3K, which includes phosphorylation of phosphatidylinositol (PI) to phosphatidylinositol 3 phosphate (PIP3), can promote the activation of protein kinase B (PKB/Akt), followed by the nuclear factor-kB (NF-kB ), resulting in over-regulation of the BCL-XL gene that favors the survival of T cells. The Activation of Akt can also promote the production of interleukin 2 (IL-2). B. The HLA-peptide complex is recognized by the TCR and by its CD4 or CD8 co-receptor, this activates the Lck kinase, which phosphorylating the CD3 complex. This leads to the recruitment and phosphorylation of the zeta-chain associated protein kinase (ZAP70), which initiates a signaling cascade that activates the phospholipase Cγ1 (PLCγ1) and RAC. PLCγ1 promotes calcium mobilization and activation of the RAS pathway. The combination of these signaling cascades promotes the activation of transcription factors and cell proliferation. C. CTLA-4 suppresses the activation and function of T cells by recruitment of the protein tyrosine phosphatase (SHP-2) and the serine/threonine phosphatase 2A protein (PP2A). These phosphatases dephosphorylate several signaling points that are essential for the co-stimulation of T cells (Modified from Alegre ML et al., 2001, Chen et al., 2013 & Nirschl et al., 2015) ^31^ ^,^ ^78^ ^,^ ^79^.

It has been proposed that CTLA-4 expression attenuates the activation of T cells by a cascade of inhibitory signals Fig. 2, as well as by its competition with CD28 ^30^. Some studies suggest that the activation of protein-tyrosine phosphatase (SHP2) and protein phosphatase 2A (PP2A) counteracts the kinase signals induced by the TCR and CD28 ^30^. Other mechanisms, including Treg cell expansion, produce immunosuppressive cytokines such as TGF-β and the enzyme IDO ^32^.

CTLA-4 blockade can affect the intratumoral immune response by inactivating Treg tumor-infiltrating lymphocytes ^33^ which can cause an increase in the Th1-dependent immune response ^4^. It has also been observed that its blockade enhances the production of specific antibodies against TAA, as well as a CD4+ cellular and CD8+ specific antigen response ^27^.

### 2.5 PD-1

PD-1 is also a key protein in immune regulation ^27^, it acts as an immune-checkpoint and immune-therapeutic target. It is a co-inhibitory molecule expressed in stimulated T cells, as well as in Treg lymphocytes, B-activated cells and NK cells ^27^ ^,^ ^31^. PD-1 appears to play a crucial role in the modulation of T cell activity through interaction with its PD-L1 and PD-L2 ligands ^31^. PD-L1 is expressed in lymphoid and non-lymphoid tissues, it is activated especially in APC, DC, macrophages and B cells, but is also expressed in tumor cells that abrogate the lymphocyte response. Expression of this ligand in tumor tissue is recognized by effector T lymphocytes, which restrict their oncolytic activity to induce cancer immunotolerance ^24^. PD-L2 is only expressed in the APC ^24^. 

After binding to its ligand, PD-1 suppresses T cell activation by recruiting SHP-2, which dephosphorylates and inactivates Zap70, an important component in the TCR signaling pathway. As result, PD-1 inhibits T-cell proliferation and its effector functions, such as the production of IFN-γ ^24^. PD-1 blockade may enhance antineoplastic immune responses by decreasing the number and suppressive activity of intratumoral Treg cells ^4^, in addition to increasing the proliferation of effector T cells (CD8+/HLA-DR+/Ki67+T cells), interferon-inducible T-cell alpha chemoattractant (I-TAC), IFN-γ and IL-18 ^4^.

### 2.6 Interacciones entre CTLA-4 y PD-1

**Figure 3:**
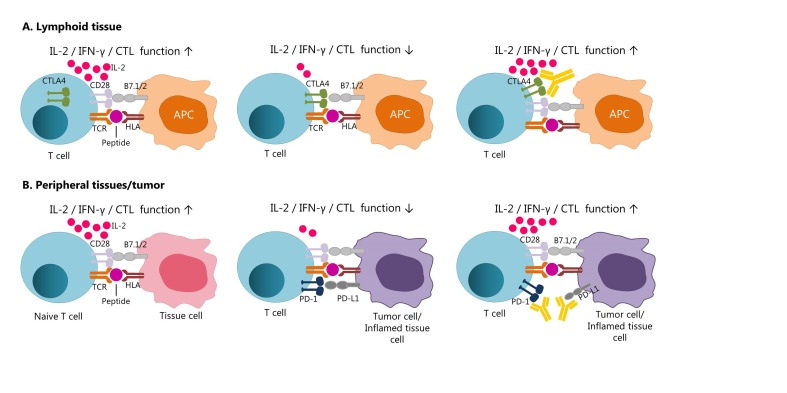
CTLA-4 and PD-1 modulate different aspects of T cell response. A) CTLA-4 is overexpressed after activation of a naïve or memory T cell in the lymphoid tissue by recognition of a specific antigen presented in the HLA context, producing a decrease in the effector function (early activation phase). The Blocking of CTLA-4 with a specific antibody would allow the signaling pathway by the CD28 receptor, contributing to the proliferation and activation of T cells. B) PD-1 is expressed primarily in memory T cells of peripheral tissues, this pathway ensures the protection of tissues from collateral damage during an inflammatory response. Tumor cells overexpress PD-1 (PD-L1 and PD-L2) ligands to evade the T-cell response against the tumor. In the same way, the use of antibodies for block the PD-1 pathway would contribute to the development of a more potent immune response. (Modified from Ott et al 2013) ^34^.

Although CTLA-4 and PD-1 negatively regulate the activation of T cells by blocking the CD3/CD28 pathway, these receptors have different roles ^27^. CTLA-4 acts during the beginning of naive and memory T cells activation in lymphoid tissue, while PD-1 operates during the effector phase of T cells Fig. 3 ^27^ ^,^ ^34^. The interaction of PD-1 with its PD-L1 ligand occurs predominantly in peripheral tissues, including tumor tissue ^15^ ^,^ ^27^ ^,^ ^34^. 

There are preclinical studies that propose a combined therapy using antibodies for the blockade of both pathways simultaneously (anti-CTLA-4 plus anti-PD-1) ^12^. This dual strategy would enhance the antitumor response but it can also be expected to be more toxic ^35^. 

### 2.7 Clinical use of immune-checkpoint blockade therapy

Ipilimumab (anti-CTLA-4) is a recombinant human monoclonal antibody (IgG1 kappa immunoglobulin) approved by the Food and Drug Administration (FDA) in 2011 for the treatment of metastatic melanoma. The human monoclonal antibody nivolumab (anti-PD-1) is an IgG4 kappa immunoglobulin authorized in Japan in 2014 for the treatment of unresectable melanoma. The FDA approved the humanized monoclonal antibody pembrolizumab (IgG4 kappa immunoglobulin) against PD-1 in September 2014 and the nivolumab in December 2014, both for the treatment of advanced melanoma. In March 2015 the FDA approved nivolumab for the treatment of lung cancer ^32^.

There are phase II studies proving increased survival in patients with metastatic melanoma who received ipilimumab ^7^ ^,^ ^36^. In one study, an average survival of 10.1 months was observed in patients using ipilimumab versus 6.4 months in patients using a control peptide vaccine ^36^; a 5-year survival rate of 18.2% was also observed in patients with advanced melanoma treated with ipilimumab+dacarbazine versus 8.8% in patients treated with placebo+dacarbazine ^37^. Two phase III studies of nivolumab showed clear benefits of this agent against metastatic melanoma compared to chemotherapy, obtaining a better survival rate at one year ^36^. In July 2017, the Bristol-Myers Squibb biopharmaceutical announced that the FDA expanded the use of intravenously administered ipilimumab as a treatment for non-extirpable metastatic melanoma in pediatric patients of 12 years or older. In addition, in August 2017, it was announced that nivolumab was approved by the FDA for the treatment of adult and pediatric patients (over 12 years of age) with metastatic colorectal cancer who present high microsatellite instability (MSI-H) or deficiencies in the repair of damaged DNA, and who had received a treatment with fluoropyrimidines, oxaliplatin, and irinotecan. The pembrolizumab and nivolumab (FDA approved) were compared with ipilimumab, demonstrating a higher response and lower toxicity ^36^. In addition, it was observed that PD-1 blockade had activity in patients who did not respond to CTLA-4 blockade ^36^. It has been proposed that agents which inhibit PD-1 are more effective than those that inhibit PD-L1 directly (e.g. human monoclonal antibody BMS-936559) ^38^ because they can inhibit both ligands (PD-L1 and PD-L2) simultaneously ^38^. 

In 2015, the safety and efficacy of nivolumab and ipilimumab were assessed separately and compared vs nivolumab+ipilimimab as a novel combined therapy (recorded as CheckMate 067 at ClinicalTrials.gov). They observed a survival of 11.4 months for the combined treatment versus 6.9 months for treatment with nivolumab alone and 2.9 months for ipilimumab alone ^39^. Although an increase of some months in the survival rate is observed, the immunotherapy has an exorbitant cost: 2015, the average cost per mg of nivolumab was estimated to $28.7, for nivolumab, $51.79 for pembrolizumab and $157.46 for ipilimumab. It should be noted that the administration dosages range from 2 mg/kg to 10 mg/kg every 3 weeks approximately. It is estimated that the cost of a patient's treatment with CheckMate 067 could reach the $295.56; the treatment with nivolumab is estimating in $103,220 and the ipilimumab in $158,252. Taking this into account, for a 75 kg patient with melanoma who wants a treatment with 26 of the highest and most frequent doses of pembrolizumab, the cost would be $ 1,009,944. If this treatment were provided to each of the 589,430 patients who die from melanoma cancer annually, the cost for the health systems would be $ 173,881,850,000. This is simply unsustainable. As in many other areas of the pharmacoeconomics of emerging drugs for chronic diseases, this represents a challenge that must be resolved by considering a balance between the demands of the community and the health systems versus the commercial interests of the entrepreneurs of the pharmaceutical industries ^40^. 

### 2.8 Adverse effects

The use of ipilimumab and tremelimumab has been associated with adverse events affecting the skin (pruritus, vitiligo), intestine (diarrhea and colitis), liver (hepatitis and elevated liver enzymes) and endocrine glands (hypothyroidism, thyroidism) 36. Compared to CTLA-4 blockade, PD-1 or PD-L1 blockade may have similar effects, but they appear to be less common 24,36. Although good results have been shown in the use of these therapies, they are not sufficiently effective to use them alone 35, which is why their combination with other strategies is necessary. Gene therapy could be an interesting alternative for combined therapy.

## 3. Suicide gene therapy

The selectivity of the antineoplastic agents is limited because cancer cells are resistant to apoptosis, cell cycle arrest, and senescence. Besides that, some resistant cells subpopulations may emerge in response to the neoplastic agent 41. The application of gene therapy could improve the selectivity of immune-checkpoint directed therapies and facilitate their access to the tumor tissue 42. Suicide therapy has two alternatives: toxin gene therapy, in which genes for a toxic protein are transduced into tumor cells, or enzyme-activating prodrug therapy. The latter has two steps: initially, a gene of a heterologous enzyme is directed and delivered to the tumor for its expression. Subsequently, a prodrug that can become a cytotoxic drug by the heterologous enzyme is administered 43. Due to its mechanism of action, this therapy triggers an anti-tumor immunoreactivity, as will be explained below.

The herpes simplex virus-thymidine kinase/ganciclovir (HSV-TK/GCV) system is selective for tumor cells because it affects the active replication of DNA, which is one characteristic of tumor cells. This activity is decreased in the surrounding stromal cells, many of which are in the quiescent state 42,43.

### 3.1 Enzymes and prodrugs used in suicide gene therapy systems

The enzymes used in suicide gene therapy are divided into two groups. The first one includes enzymes of non-mammalian origin (e.g. HSV-TK). The second comprises enzymes of human origin that are absent or subexpressed in tumor cells ^43^. Several enzyme-prodrug systems have been developed for suicide gene therapy, such as the carboxyl esterase (CE)/irinotecan, carboxypeptidase A (CPA)/MTX-a-peptide, carboxypeptidase G2 (CPG2)/CMDA and HSV-TK/GCV. The HSV-TK/GCV system is the most studied and has progressed successfully to advanced phases in clinical trials, which is explained in more detail below ^42^ ^,^ ^44^.

### 3.2 HSV-TK/GCV system

**Figure 4:**
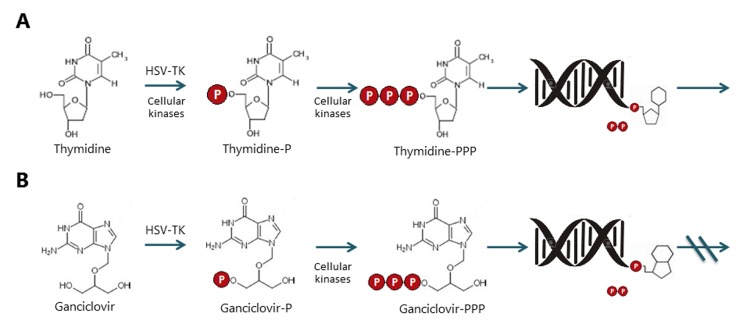
Comparative metabolism of the thymidine and ganciclovir by Herpes simplex virus thymidine kinase and cellular kinases. A. Thymidine metabolism. Typically, thymidine kinase enzymes can phosphorylate the thymidine to thymidine triphosphate, for further integration into DNA. B. Metabolism of ganciclovir. The HSV thymidine kinase, unlike to human thymidine kinase, is able to phosphorylate ganciclovir to convert to GCV-P, once this happened, the cellular kinases can phosphorylate it for later integration into the DNA, which leads to the arrest of its synthesis and therefore, the cell death.

The HSV-TK/GCV system uses ganciclovir and its analogs as prodrugs. These are analogs of purine nucleosides ^5^ ^,^ ^42^. Systemic administration of GCV induces selective apoptosis in cells transduced with the TK gene. HSV-TK is able to phosphorylate the GCV, turning it into monophosphorylated GCV, which is subsequently tri-phosphorylated by cellular kinases. This product blocks the DNA replication, causing its fragmentation and apoptosis ^45^ ^,^ ^46^ Fig. 4.

The HSV-TK/GCV system has been tested in pre-clinical ^47^ ^,^ ^48^ and clinical studies against several types of cancer, such as prostate ^49^ ^,^ ^50^, brain ^51^ ^-^ ^53^, ovarian ^54^ ^,^ ^55^, bladder ^56^ ^,^ ^57^, cervix ^58^, pancreatic ^59^ ^,^ ^60^ and liver cancer ^61^, among others. Several phase I and II clinical trials have demonstrated the safety and efficacy of this therapy in humans ^43^ ^,^ ^44^. Other studies have shown that HSV-TK gene transduction is safer and more effective with adenoviral replication deficient vectors compared to retroviral vectors ^51^ ^,^ ^62^.

### 3.3 Immune response induced by the AdV-TK/GCV system 

It has been shown that HSV-TK/GCV therapy induces anti-tumor immunity ^63^ ^,^ ^64^ and even regression of brain tumors in immunocompetent rats and neuroblastoma in a murine model ^65^ ^,^ ^66^. In the treated tumors, a marked infiltration of inflammatory cells, predominantly CD4+ and CD8+, is observed. This suggests that the HSV-TK/GCV system stimulates the antitumor immune response ^50^ ^,^ ^60^. The overexpression of different costimulatory molecules such B7.1 and B7.2, intracellular adhesion molecules (ICAM) and MHC molecules, and the attraction and activation of APCs are also observed in tumor tissue ^67^ ^,^ ^68^.

During the HSV-TK/GCV tumor treatment, the levels of some cytokines that stimulate APCs and T cells, such as IL-2, IL-12, IFNγ, TNFα and GM-CSF, are increased; whereas inhibitory cytokines like IL- 4, IL-6 and IL-10 are not stimulated ^67^. The increase in the immune response has been demonstrated by higher levels of circulating active CD8 cells and elevated IL-12 in serum ^69^ ^,^ ^70^, a key mediator of the cellular immune response against viral infections and malignant tumors ^69^ ^,^ ^71^. Interestingly, NK cell levels have been linked to IL-12 levels, because NK cells are one of the targets of this interleukin ^69^ ^,^ ^72^.

The viral TK protein also functions as a superantigen, stimulating a highly immunogenic tumor microenvironment ^68^. This protein induces the release and presentation of TAA which can be recognized by T lymphocytes and therefore generate an adaptive immune response. This can lead to tumor cell cytolysis and posterior recruitment of APCs ^68^. The activated APCs induce T cells proliferation by the secretion of IL-2 and IL-12 at the tumor site. All these events are desirable to get a powerful anti-tumor effect ^73^ ^,^ ^74^. However, a contradictory observation should be considered. A clinical trial of HSV-TK/GCV as neoadjuvant therapy for pancreatic carcinoma using an adenoviral vector showed increased intratumoral levels of PD-L1 in samples analyzed after surgical resection. This event may decrease the effector T cell response but may be reversed with co-administration of PD-1 / PD-L1 inhibitors ^60^. 

The viral vector-mediated HSV-TK/GCV therapy has shown effective progression to phase III in some clinical trials when it was used alone or in combination with chemotherapy or radiation for prostate cancer, hepatocellular carcinoma, or glioblastoma multiforme ^67^ ^,^ ^68^ ^,^ ^75^.

### 3.4 Adverse effects

Despite the promising results, there are some disadvantages. The replication-deficient adenoviral vectors trigger a strong humoral and cellular immune response that limits its effectiveness to a period of two to three weeks. Regardless these vectors may be useful for therapeutic applications where a very high level of transient expression of the therapeutic gene is desirable, as would occur in cancer gene therapy ^76^. On the other hand, the expression of the TK protein is not tumor specific. An interesting option is the use of adenoviruses that prefer their replication in tumor cells using a specific promoter ^75^.

Phase I and II studies have shown some side effects, such as mild fever, neutropenia, headache, thrombocytopenia, and impaired hepatic enzymes, among others. Fortunately, these events are transient and easy to tolerate ^49^ ^,^ ^51^ ^,^ ^62^.

## 4. Combined therapy as a new treatment

The combined treatment of gene therapy and immunotherapy is an attractive option that recent advances in cancer therapeutics have made possible. The use of a suicide gene therapy system would lead to the sudden and massive presentation of TAA over a sustained period of weeks or months. It is reasonable to think that this therapy can be synergistically enhanced by its combination with a systemically administered immune-checkpoint inhibitor drug such as those described in this review. To explain this idea in a better way, it could be assumed that a tumor treated with HSV-TK/GCV will generate sudden and massive exposure of TAA to the immune system, which in other conditions wouldn't generate an effective immunoreactivity due to the decrease of MCH I and costimulating molecules and by the induction of Treg. This event would trigger the attraction and activation of APC and some TAA could be expected to induce an increase in the number of intratumoral Treg lymphocytes (even before the intervention with gene therapy), leading to an immunosuppressive environment. In order to prevent this immunological phenomenon and to enhance the antitumor response, subsequent administration of an immune-checkpoint inhibitor, for example an anti-PD1 antibody, would affect the activity of intratumoral Treg cells. This would lead to an increase in the proliferation of the effector T cells capable of fighting the tumor and would reinforce an immune memory response that would potentially have a long-term protective effect ^36^. 

Recently performed tests with adenoviral vectors that carry the HSV-TK gene and the PD-1 extracellular domain sequence fused to the Fc portion of mouse IgG2a was recently assayed to produce the soluble PD-1 (sPD1-Ig) segment. This segment inhibits activity of the complete ligand competitively, and consequently, inhibits the apoptotic effect of T cells mediated by the immunosuppressive interaction of the whole ligand with its receptor. This vector was administered in a murine model of colon carcinoma and demonstrated a synergy between HSV/TK therapy and competitive blockade of PD-1/PD-L1 binding. There was a significant decrease in tumor volume in the group of mice treated with HSV-TK/sPD1 compared to the control groups, including the group of mice treated with the simple scheme of HSV-TK / GCV ^77^. 

 It is possible that in the near future, preclinical and clinical trials will continue to test hypotheses similar to the one proposed in this section and will certainly have very effective clinical results, and above all, with a high level of therapeutic selectivity, which will favor even more the development of the precision medicine in the area of oncology.

## Conclusion

The increase of the immune response against tumors could be a key strategy to fight against cancer. The TK/GCV system induces the massive presentation of TAA effectively. On the other hand, the expression of the TK super-antigen facilitates antitumor cellular immunity. The use of monoclonal antibodies against the immune-checkpoints, such as CTLA-4 and PD-1, can decrease the tumor immunosuppression. Until now both strategies are found in clinical trials and have shown promising results. It would be expected that the combination of these two types of therapies would be synergistic, more selective and effective and would have a long-term protective effect.
