# Supplementary material for: Immunotherapy and gene therapy as novel treatments for cancer
Source: Colomb Med (Cali). 2017 Sep 30;48(3):138–47. doi: 10.25100/cm.v48i3.2997 (PMC5687866; doi:10.25100/cm.v48i3.2997)
Supplement: Translation

# Translation: Inmunoterapia y terapia génica como nuevos tratamientos contra el cáncer

## Introducción

La progresión de muchos tipos de cáncer se acompaña de una fuerte supresión del sistema inmune (SI), lo que interfiere con la respuesta antitumoral y la erradicación eficaz del tumor ^1^. La evasión de la inmuno-vigilancia es atribuida, en parte, a que el microambiente tumoral puede inhibir la proliferación de linfocitos T y atraer a poblaciones celulares supresoras ^2^. 

La mejor comprensión del sistema inmunológico del tumor ha permitido el desarrollo de terapias específicas diseñadas para mejorar la respuesta inmune de un paciente. La inmunoterapia tumoral tiene dos estrategias: atacar al tumor directamente o activar el SI usando terapias celulares, como la estimulación con agonistas o el bloqueo de puntos de control inmunológicos ^3^. Este último libera el potencial de la respuesta inmune antitumoral, mostrando ser una terapia prometedora ^4^. Otra opción antitumoral es el uso de modalidades de terapia génica, que permiten modificar la expresión génica con propósitos terapéuticos. Por ejemplo, ésta puede facilitar la transducción de células tumorales con "genes suicidas", una de las estrategias de terapia génica antitumoral más investigadas ^5^. 

En este artículo se hace una revisión del uso de terapia génica suicida y el bloqueo de los puntos de control inmunológicos como alternativas para el tratamiento del cáncer y se analizan los posibles efectos sinérgicos que podrían lograrse con la combinación de estas dos terapias. 

## 1. Cáncer y el sistema inmune

El SI interactúa íntimamente con los tumores durante el proceso del desarrollo de la enfermedad y su progresión a metástasis (inmunología tumoral) ^6^. El SI responde al cáncer, reconociendo y eliminando las células anormales (inmuno-vigilancia) ^7^. Sin embargo, algunas células resistentes escapan de este control (inmunoedición) ^8^ disminuyendo su inmunogenicidad ^9^, promoviendo el crecimiento maligno ^7^. 

Las células tumorales presentan cambios en sus marcadores de superficie. Por ejemplo, expresan antígenos asociados a tumor (TAA) ^10^ o disminuyen la expresión del complejo mayor de histocompatibilidad (MHC) de clase I. Esto puede activar células de la respuesta inmune innata, como las células asesinas naturales (NK) ^10^. Los macrófagos y neutrófilos atacan las células tumorales y estimulan a los linfocitos T citotóxicos (CTL), a las células presentadoras de antígeno (APC) y a las NK. En contraste, las células inflamatorias producen factores de crecimiento tumorales (TGF) y factores estimuladores de la angiogénesis que contribuyen al desarrollo del tumor ^11^. 

En la respuesta adaptativa, los TAA procesados son presentados por moléculas MHC clase I y II de las APC a los receptores específicos para la activación de las células T CD8+ o CD4+, respectivamente ^10^^,^^12^. Los linfocitos T CD8+ son consideradas las principales células efectoras antitumorales ^13^. Una vez que son activadas, median la lisis de las células cancerosas ^10^. Dentro de las células T CD4+, las Th1 son responsables de la inmunidad celular: secretan interleucina (IL)-2, TNFα e interferón-γ (IFN-γ), promueven la actividad citotóxica de los macrófagos e inducen la sobreexpresión de MHC I y II en las APC. En contraste, las células Th2 expresan IL-4,- 5, -10 y -13, induciendo anergia clonal, potenciando la inmunidad humoral y regulando la actividad de los macrófagos ^13^. Por otro lado, los linfocitos T reguladores (Treg), ayudan a reducir la inflamación produciendo TGF-β, IL-35 e IL-10 ^10^. Las células tumorales pueden secretar quimiocinas como CCL22 para reclutar células Treg con el objetivo de suprimir la función efectora de las células T y disminuir la respuesta inmune ^10^.

Los tumores también pueden desregular el SI alterando un complejo balance entre señales inhibitorias (puntos de control) y activadoras en diferentes vías que regulan la acción de las células T ^7^. 

## 2. Linfocitos T reguladores

Los linfocitos Treg son relevantes para el mantenimiento de la homeostasis inmunológica: mantienen la tolerancia a antígenos propios, evitan la aparición de enfermedades autoinmunes, controlan el desarrollo de una respuesta inmune y favorecen el escape de las células tumorales del control inmunológico ^14^^,^ ^15^ . La subpoblación mejor caracterizada expresa CD4, CD25 y Foxp3. Las poblaciones celulares que son suprimidas por los linfocitos Treg incluyen a los linfocitos T CD4+ y CD8+, las células T naturales asesinas T (células NKT), las células dendríticas (DCs), los monocitos/macrófagos, los linfocitos B y las células NK ^14^. 

Debido a que los Treg suprimen la respuesta inmune contra antígenos propios ^8^^,^^16^, se postula que los TAAs pueden inducir un incremento en el número de linfocitos Treg intratumorales en varias neoplasias, incluyendo el cáncer colorrectal (CRC), facilitando la inmunotolerancia tumoral ^8^^,^^17^. La acumulación de Treg en tumores se explica por varios mecanismos, como la conversión de células T CD4+ a Treg en respuesta a TGF-β de membrana, el reclutamiento de Tregs por las quimiocinas CCL17, CCL22 y CCL28 o la secreción tumoral de VEGF-A en respuesta a la hipoxia, lo cual inhibe la maduración de DC. Las DC inmaduras expresan TGF-β favoreciendo la conversión de células T CD4+ a Treg ^18^.

Los TAA más frecuentes son antígenos propios poco expresados en células normales, pero altamente expresados en las tumorales ^18^. El antígeno carcinoembrionario (CEA) expresado altamente en CRC ^18^, uno de los mejor conocidos, es reconocido como antígeno propio por los Tregs ^19^, ocasionando una respuesta inmune deficiente frente a las células tumorales. En cánceres de ovario, mama, páncreas, estómago e hígado, un incremento de linfocitos Treg en el tumor se asocia a empeoramiento del pronóstico^17^. El uso de estas células como blanco puede impactar las estrategias terapéuticas contra el cáncer ^8^. 

### 2.1 Mecanismos de acción de los linfocitos Treg

Los linfocitos Treg tienen cuatro mecanismos principales de acción para regular la respuesta inmune Fig. 1. El primero es la supresión por citocinas inhibitorias, las cuales incluyen IL-3, IL-10 y TGFβ ^18^^,^^20^. El segundo es la supresión por citólisis. Los Treg pueden tener inducir citólisis de las células B por medio de la producción de granzima B. Estas células también pueden ejercer un efecto citolítico en linfocitos T CD8+ y células NK mediado por granzima B y perforina, o por la vía aTRAIL-DR5 ^20^^-^^22^. El tercero es la supresión por alteraciones metabólicas que afectan la actividad de moléculas como CD25 (receptor de IL-2), AMP cíclico (cAMP), CD39, CD73 y el receptor de adenosina 2A (A2AR) ^20^. El cuarto mecanismo consiste en la supresión de la maduración o de la función de DC. Esto incluye vías como la del gen 3 de activación de linfocitos (LAG3) o la interacción entre el antígeno asociado a los linfocitos T citotóxicos (CTLA-4) y CD80/86, la cual induce a la enzima indoleamina-2,3-dioxigenasa (IDO), una molécula inmunosupresora generada por las DC ^10^^,^^20^. El bloqueo de estos mecanismos inmunosupresores podría incrementar la función de las células T y generar una respuesta clínica más efectiva ^8^^,^^20^.

**Figura 1:** Mecanismos de acción de las células Treg. A. Producción de citocinas inhibitorias como IL-10, IL-35, y TGF-β. B. La inhibición por citólisis incluye mecanismos dependientes de granzima A o granzima B, así como dependientes de perforina. C. Se puede dar una interrupción metabólica por parte de las células Treg al sobre-producir CD25, capturando la IL-2; realizar una inhibición por cAMP o inmunosuprimir por medio del receptor de adenosina 2A. D. Se puede modular la maduración o función de las DC por medio de las vías LAG3, CTLA-4 o por la enzima IDO (Modificado de Vignali *et al*. 2008) ^20^.

### 2.2 Regulación de los puntos de control inmunológicos por medio de linfocitos Treg

En el caso de las células T, la amplitud y calidad de la respuesta después del reconocimiento de un antígeno, está regulada por un balance entre señales coestimuladoras e inhibitorias (puntos de control inmunológico) ^4^. Con el objetivo de aumentar la función efectora de las células T infiltrantes de tumor, se pueden inhibir las señales inmunosupresoras; de esta manera, se ha tenido un mayor éxito clínico en el tratamiento de varios tipos de cáncer, como melanoma y cáncer de pulmón ^6^. Las moléculas que actúan como puntos de control inmunológico, como el receptor CTLA-4 y la proteína de muerte celular programada 1 (PD-1), son expresadas en los linfocitos Treg y en las células T efectoras infiltrantes de tumor ^15^. 

### 2.3 Puntos de control inmunológico en cáncer 

Como se comentó previamente, la activación e inhibición de diferentes receptores regula el balance entre la respuesta y la tolerancia inmune ^23^^,^^24^, siendo estos importantes para una completa activación y función efectora de las células T ^25^. La terapia con anticuerpos contra reguladores inmunológicos negativos ha demostrado éxito en el tratamiento antineoplásico ^23^^,^^25^, debido a que aumenta el potencial de la respuesta inmune antitumoral. Está demostrado que los tumores usan algunas vías de control inmunológico como mecanismo de resistencia inmune ^4^, ya que al incrementarse la expresión de las proteínas de los puntos de control, decrece la función de las células T. Ejemplos de puntos de control inmunológicos son PD-1, CTLA-4, LAG-3, inmunoglobulina de célula T (TIM-3), entre otros ^26^.

Los anticuerpos antitumorales que bloquean los puntos de control inmunológico están dirigidos contra receptores de linfocitos o sus ligandos ^27^ Tabla 1. Dos puntos de control inmunológicos ampliamente estudiados en el contexto clínico de la inmunoterapia contra el cáncer son CTLA-4 y PD-1; ambos son receptores inhibitorios que regulan la respuesta inmune ^27^. Aunque la inhibición de los puntos de control parece ser exitosa en el tratamiento de algunos cánceres, está asociada eventos adversos, en particular a respuestas autoinmunes que afectan a órganos como el colon, la piel, algunas glándulas endócrinas, el hígado, etc ^26^.

**Tabla 1:** Desarrollo de agentes farmacológicos dirigidos contra vías de señalización de inmuno-checkpoints ^36^^,^^80^.

Blanco	Función biológica	Anticuerpo	Situación en clínica
CTLA-4	Receptor inhibitorio	Ipilimumab	Aprobado por la FDA para melanoma. Ensayos de fase IV para melanoma y cáncer metastásico de células renales. Ensayos de fase III para cáncer de estómago/esófago, cáncer de pulmón de células pequeñas y no pequeñas, carcinoma renal, mesotelioma pleural, carcinoma metastásico de células escamosas de cabeza y cuello, cáncer de próstata, melanoma ocular.
		Tremelimumab	Probado en ensayos de fase III para melanoma, cáncer de cabeza y cuello, cáncer de células de pulmón pequeñas y no pequeñas, cáncer urotelial,
PD-1	Receptor inhibitorio	Nivolumab	Aprobado por la FDA para melanoma, carcinoma renal, cáncer de células de pulmón no pequeñas. Ensayos de fase IV para carcinoma renal metastásico avanzado y melanoma metastásico. Ensayos fase III para cáncer de pulmón de células pequeñas y no pequeñas, cáncer de estómago/esófago, melanoma, mesotelioma, carcinoma hepatocelular, mieloma múltiple, cáncer urotelial, cáncer gástrico
		Pembrolizumab	Aprobado por la FDA para melanoma, cáncer de células de pulmón no pequeñas. Ensayos de fase III para melanoma y cáncer de pulmón de células pequeñas y no pequeñas.
		Pidilizumab	Ensayos de fase I/II para linfoma, mieloma múltiple, cáncer de páncreas.
PDL1	Ligando de PD-1	BMS-936559	Ensayos de fase I para melanoma.
		Atezolizumab	Ensayos de fase III para cáncer de pulmón de células pequeñas y no pequeñas, cáncer de mama triple negativo, cáncer de tracto urinario, cáncer renal, cáncer de ovario, cáncer colorrectal, melanoma.
LAG3	Receptor inhibitorio	IMP321	Ensayos de fase I/II para adenocarcinoma de mama, carcinoma renal, melanoma, neoplasias pancreáticas.
B7-H3	Ligando inhibitorio	Enoblituzumab	Ensayos de fase I para vários tipos de cáncer.

CTLA-4: antígeno asociado a linfocitos T citotóxicos

LAG3: gen 3 de activación a linfocitos

PD-1: receptor proteíco de muerte celular programada 1

PDL1: ligando de PD-1

### 2.4 CTLA-4

El receptor CTLA-4 es el primer punto de control inmunológico usado como blanco clínico ^28^. CTLA-4 es miembro de la súper-familia de las inmunoglobulinas CD28:B7, normalmente es expresado en bajos niveles en la superficie de las células T efectoras y células Treg. Su función es regular la amplitud de las primeras etapas de la activación de estas células ^28^. Para que una célula T se active se requieren tres señales: la unión del antígeno con el receptor de células T (TCR), la interacción del MHC (en humanos antígeno leucocitario humano, HLA) con los receptores CD8 o CD4 de las células T y la generación de una señal coestimuladora generada por la unión de CD80(B7) con CD28 ^12^^,^^29^. Una vez que esto ocurre, la vía de CD28 amplifica la señalización del TCR para activar la proliferación de las células T. CD28 y CTLA-4 comparten los mismos ligandos: CD80(B7.1) y CD86(B7.2), aunque estos se unen a CTLA-4 con mayor afinidad ^4^^,^^30^, debido a esto, CTLA-4 contrarresta la actividad coestimuladora de CD28 ^4^^,^^31^. 

La importancia de CTLA-4 en la activación de células T está demostrada por el fenotipo letal del sistema inmune hiperactivado en ratones knockout para CTLA-4^4^. A pesar de que CTLA-4 se expresa en células efectoras T CD8+ activadas, su función fisiológica más importante se ejerce a través de distintos efectos en los dos subconjuntos principales de células T CD4+: la disminución de la actividad de las células T cooperadoras (Th1) y el potenciamiento de la actividad inmunosupresora de las células Treg ^4^.

Se ha propuesto que la expresión de CTLA-4 atenúa la activación de las células T por una cascada de señales inhibitorias Fig. 2, así como por su competencia con CD28 ^30^. Algunos estudios sugieren que la activación de las proteína tirosina fosfatasa (SHP2) y de la proteína fosfatasa 2A (PP2A) contrarrestan las señales de las quinasas inducidas por TCR y CD28 ^30^. Otros mecanismos, incluyen la expansión de células Treg, estas producen citocinas inmunosupresoras como TGF-β y la enzima IDO^32^.

**Figura 2:** Modelo de señalización de CD28 y CTLA-4. A. Cuando ocurre la estimulación de células T, se fosforilan los residuos intracelulares de tirosina de CD28, esto atrae a la quinasa 3 fosfatidilinositol (PI3K). La activación de PI3K, la cual incluye la fosforilación del fosfatidilinositol (PI) a fosfatidilinositol 3 fosfato (PIP3), puede promover la activación de la proteína quinasa B (PKB/Akt), seguida de la del factor nuclear-kB (NF-kB), resultando en la sobreregulación del gen BCL-XL que favorece la supervivencia de las células T. La activación de Akt puede también promover la producción de interleucina 2 (IL-2). B. El complejo HLA-péptido es reconocido por el TCR y por su co-receptor CD4 o CD8, lo que activa a la quinasa Lck, la cual fosforila al complejo CD3. Esto ocasiona el reclutamiento y fosforilación de la proteína asociada a la cadena ζ (ZAP70), la cual inicia una cascada de señalización que activa la fosfolipasa Cγ1 (PLCγ1) y RAC. PLCγ1 promueve la movilización de calcio y la activación de la vía RAS. La combinación de estas cascadas de señalización promueve la activación de factores de transcripción y proliferación celular. C. CTLA-4 suprime la activación y la función de las células T mediante el reclutamiento de la proteína tirosina fosfatasa (SHP-2) y la proteína serina/treonina fosfatasa 2A (PP2A). Estas fosfatasas desfosforilan varios de los puntos de señalización que son esenciales para la co-estimulación de las células T (Modificado de Alegre ML *et al*. 2001, Chen et al. 2013 & Nirschl *et al*. 2015) ^31^^,^^78^^,^^79^.

El bloqueo de CTLA-4 puede afectar la respuesta inmune intratumoral mediante la inactivación de los linfocitos Treg infiltrantes de tumor ^33^ y ocasionar un incremento en la respuesta inmune dependiente de células Th1 ^4^. También se ha observado que su bloqueo potencializa la producción de anticuerpos específicos contra TAA, así como una respuesta celular CD4+ y CD8+ antígeno específica ^27^.

### 2.5 PD-1

PD-1 también es una proteína clave en la regulación inmune ^27^, actúa como un punto de control inmunológico y es un blanco inmuno-terapéutico. Es una molécula co-inhibitoria expresada en células T estimuladas, así como en los linfocitos Treg, células B-activadas y células NK ^27^^,^^31^. PD-1 parece tener una función crucial en la modulación de la actividad de las células T a través de la interacción con sus ligandos PD-L1 y PD-L2 ^31^. PD-L1 se expresa en tejidos linfoides y no linfoides, es activado especialmente en APCs, DCs, macrófagos y células B, aunque también se expresa en las células tumorales que abrogan la respuesta linfocitaria. La expresión de este ligando en el tejido tumoral es reconocida por los linfocitos T efectores, los cuales restringen su actividad oncolítica para inducir inmunotolerancia al cáncer ^24^. PD-L2 solo se expresa en las APCs ^24^. 

Después de la unión con su ligando, PD-1 suprime la activación de células T reclutando SHP-2, la cual desfosforila e inactiva Zap70, un componente importante en la vía de señalización del TCR. Como resultado, PD-1 inhibe la proliferación de células T y sus funciones efectoras, como la producción de IFN-γ ^24^. El bloqueo de PD-1 puede mejorar las respuestas inmunes antineoplásicas al disminuir el número y actividad supresora de las células Treg intratumorales ^4^, además de incrementar la proliferación de células T efectoras (CD8+/HLA-DR+/Ki67+células T), la producción del quimioatrayente alfa de células T inducible con interferón (I-TAC), de IFN-γ y de IL-18 ^4^.

### 2.6 Interacciones entre CTLA-4 y PD-1

Aunque CTLA-4 y PD-1 regulan de manera negativa la activación de las células T al bloquear la vía CD3/CD28, estos receptores tienen distintos papeles ^27^. CTLA-4 opera durante el inicio de la activación de las células T nativas y de memoria en tejido linfoide y PD-1 opera durante la fase efectora de las células T Fig. 3 ^27^^,^^34^. La interacción de PD-1 con su ligando PD-L1 ocurre predominantemente en los tejidos periféricos, incluyendo el tejido tumoral ^15^^,^^27^^,^^34^. 

**Figura 3:** CTLA y PD-1 modulan diferentes aspectos de la respuesta de las células T. A) CTLA-4 es sobreexpresado después de la activación de una célula T virgen o de memoria en tejido linfático por el reconocimiento de un antígeno especifico presentado en el contexto de HLA, ocasionando un decremento en la función efectora (fase de activación temprana). El bloqueo de CTLA-4 con un anticuerpo específico permitiría la vía señalización por el receptor CD28, contribuyendo a la proliferación y activación de células T. B) PD-1 es expresado principalmente en células T de memoria de tejidos periféricos, esta vía asegura la protección de los tejidos del daño colateral durante una respuesta inflamatoria. Las células tumorales sobreexpresan los receptores de PD-1 (PD-L1 y PD-L2) para evadir la respuesta de las células T hacia el tumor. De la misma manera, al utilizar anticuerpos para bloquear la vía de PD-1 se contribuiría al desarrollo de una respuesta inmune más potente. (Modificada de Ott *et al*. 2013) ^34^.

Existen estudios preclínicos que proponen una terapia combinada utilizando anticuerpos para el bloqueo simultáneo de ambas vías (anti-CTLA-4 más anti-PD-1) ^12^. Esta estrategia dual potenciaría la respuesta antitumoral, pero también se puede prever que resulte más tóxica ^35^. 

### 2.7 Uso clínico del bloqueo de puntos de control inmunológicos

El ipilimumab (anti-CTLA-4) es un anticuerpo monoclonal recombinante humano (inmunoglobulina IgG1 kappa) aprobado por la *Food and Drug Administration* (FDA) en el 2011 para el tratamiento de melanoma metastásico. El anticuerpo monoclonal humano nivolumab (anti-PD-1) es una inmunoglobulina IgG4 kappa autorizada en Japón en el 2014 para el tratamiento del melanoma no resecable. La FDA aprobó el anticuerpo monoclonal humanizado pembrolizumab (inmunoglobulina IgG4 kappa) contra PD-1 en septiembre de 2014 y el nivolumab en diciembre de 2014, ambos para el tratamiento de melanomas avanzados. En marzo del 2015 la FDA aprobó el nivolumab para el tratamiento de cáncer de pulmón ^32^.

Existen estudios de fase II que muestran una mayor sobrevida en pacientes con melanoma metastásico a los que se les administró ipilimumab ^7^^,^^36^. En un estudio, se observó una sobrevida media de 10.1 meses en pacientes que usaron ipilimumab contra 6.4 meses en pacientes que usaron una vacuna peptídica control ^36^; también se observó una tasa de supervivencia en 5 años del 18.2% para pacientes con melanoma avanzado tratados con ipilimumab+dacarbazina contra el 8.8% de pacientes tratados con placebo+dacarbazina ^37^. Dos estudios fase III de nivolumab establecieron claros beneficios de este agente contra melanoma metastásico en comparación con la quimioterapia, mostrando una mejor tasa de supervivencia a un año ^36^. En julio del 2017, la compañía Bristol-Myers Squibb anunció que la FDA expandió el uso de ipilimumab administrado de manera intravenosa como tratamiento para melanoma metastásico no extirpable en pacientes pediátricos de 12 años de edad o mayores. Así mismo, en agosto del 2017, anunció que el nivolumab fue aprobado por la FDA para el tratamiento de pacientes adultos y pediátricos (mayores de 12 años) con cáncer colorrectal metastásico que presentan alta inestabilidad microsatelital (MSI-H) o deficiencia en la reparación de errores en el DNA, en los pacientes refractarios al tratamiento con fluoropirimidinas, oxaliplatino e irinotecan. El pembrolizumab y el nivolumab (aprobados por la FDA) fueron comparados contra el ipilimumab, demostrándose una mayor respuesta y una menor toxicidad ^36^. Además se observó que el bloqueo de PD-1 tuvo actividad en pacientes que no respondieron al bloqueo de CTLA-4 ^36^. Se ha propuesto que los agentes que inhiben PD-1 son más efectivos que los que inhiben directamente a PD-L1 (ej. anticuerpo monoclonal humano BMS-936559 ^38^, debido a que pueden inhibir ambos ligandos (PD-L1 y PD-L2) de manera simultánea ^38^. 

En 2015 se evaluó la seguridad y eficiencia del nivolumab e ipilimumab por separado y en comparación con una terapia combinada nivolumab + ipilimimab (registrado como CheckMate 067 en ClinicalTrials.gov), observándose una sobrevida de 11.4 meses para el tratamiento combinado, contra con 6.9 meses para el tratamiento solo con nivolumab y 2.9 meses para el ipilimumab ^39^. Aunque se observa un aumento en la supervivencia de algunos meses, la inmunoterapia tiene un costo exorbitante; en el 2015 se estimó un costo promedio por mg de $28.78 dólares para el nivolumab, $51.79 dólares para el pembrolizumab y $157.46 dólares para el ipilimumab. Hay que considerar que las dosis de administración van desde 2 mg/kg hasta 10 mg/kg cada 3 semanas, aproximadamente. Se calcula que el costo del tratamiento de un paciente con CheckMate 067 podría ser de $295,566 dólares; el del nivolumab de $103,220 dólares y del ipilimumab de $158,252 dólares. Teniendo en cuenta lo anterior, para un paciente de 75 kg con melanoma que recibe 26 de las dosis más altas y frecuentes de pembrolizumab, el costo del tratamiento sería de $1,009,944 dólares. Si el tratamiento se le proporcionara a cada uno de los 589,430 pacientes que mueren por cáncer de melanoma anualmente, el costo para los sistemas de salud en general sería de $173,881,850,000 dólares. Esto es simplemente insostenible. Como en muchas otras áreas de la famacoeconomía de los medicamentos emergentes para las enfermedades crónicas, lo anterior representa un reto que debe resolverse considerando un equilibrio entre las demandas de la comunidad y de los sistemas de salud versus los intereses comerciales de los empresarios de las industrias farmacéuticas ^40^. 

### 2.8 Efectos adversos

El uso de ipilimumab y tremelimumab se ha asociado con eventos adversos que afectan la piel (prurito, vitíligo), el intestino (diarrea y colitis), el hígado (hepatitis y enzimas hepáticas elevadas) y las glándulas endócrinas (hipotiroidismo, tiroidismo) ^36^. En comparación con el bloqueo de CTLA-4, el bloqueo de PD-1 o PD-L1 puede tener efectos similares, pero parecen ser menos comunes ^24^^,^^36^. Aunque se han mostrado buenos resultados en el uso de estas terapias, no son lo suficientemente efectivas para utilizarse solas ^35^, por lo que es necesaria su combinación con otras estrategias. La terapia génica podría ser una alternativa interesante para una terapia combinada.

## 3. Terapia génica suicida

La selectividad terapéutica de los agentes antineoplásicos es limitada, porque las células cancerosas son resistentes a la apoptosis, al arresto del ciclo celular y a la senescencia, además que pueden emerger subpoblaciones celulares resistentes ^41^. La aplicación de tecnologías de terapia génica podría mejorar la selectividad de las terapias dirigidas a los puntos de control inmunológico y facilitar el acceso de estas al tejido tumoral ^42^. La terapia suicida tiene dos alternativas: la terapia génica tóxica, en la cual se transducen genes para una proteína tóxica en las células tumorales, o la terapia de activación enzimática de profármacos. Esta última consta de dos etapas: inicialmente, el gen de una enzima heteróloga es dirigido y entregado al tumor para su expresión. Posteriormente, se administra un profármaco que es convertido en un medicamento citotóxico por la enzima heteróloga ^43^. Debido a su mecanismo de acción, esta terapia confiere una inmunorreactividad antitumoral, como se explicará más adelante.

El sistema HSV-TK/GCV es selectivo para las células tumorales debido a que afecta la replicación activa del DNA de manera preferencial en las células tumorales, actividad que es baja en las células estromales circundantes, muchas de las cuales están en estado quiescente ^42^^,^^43^.

### 3.1 Enzimas y profármacos usados en sistemas de terapia génica suicida

Las enzimas utilizadas en terapia génica suicida se dividen en dos grupos. El primero comprende enzimas de origen no mamífero (ej. timidina quinasa del virus *Herpes simplex [HSV-TK]*. El segundo comprende enzimas de origen humano que están ausentes o se expresan a bajas concentraciones en células tumorales ^43^. Se han desarrollado varios sistemas enzima-profármaco para terapia génica suicida, como los sistemas carboxil esterasa (CE)/irinotecan, carboxipeptidasa A (CPA)/péptidos MTX-a, carboxipeptidasa G2(CPG2)/CMDA y HSV-TK/ganciclovir (GCV). Este último sistema es el más estudiado y ha progresado exitosamente a ensayos clínicos de fases avanzadas, por lo cual se explica con más detalle a continuación ^42^^,^^44^.

### 3.2 Sistema HSV-TK/GCV

El sistema HSV-TK/GCV utiliza al ganciclovir y sus análogos como profármacos. Estos profármacos son análogos de los nucleósidos de purina ^5^^,^^42^. La administración sistémica del GCV induce apoptosis selectiva en las células transducidas con el gen *TK*. La HSV-TK es capaz de fosforilar el GCV, convirtiéndolo en GCV monofosforilado, que posteriormente es tri-fosforilado por quinasas celulares. Este producto bloquea la replicación del DNA, causando además su fragmentación y apoptosis ^45^^,^^46^ Fig. 4.

**Figura 4:** Metabolismo comparativo de la timidina y del ganciclovir por la timidina quinasa del virus *Herpes simplex* y las quinasas celulares. A. Metabolismo de la timidina. De manera normal, las enzimas timidina quinasa pueden fosforilar la timidina hasta obtener timidina trifosfato, para su posterior integración al DNA. B. Metabolismo del ganciclovir. La timidina quinasa del HSV, a diferencia de la timidina quinasa humana, es capaz de fosforilar el ganciclovir para convertirlo en GCV-P, una vez que esto ocurre, las quinasas celulares pueden fosforilarlo para su posterior integración en el DNA, esto ocasiona el arresto de su síntesis y por consiguiente, la muerte de la célula.

El sistema HSV-TK/GCV se ha ensayado en estudios pre-clínicos ^47^^,^^48^ y clínicos contra varios tipos de cáncer, como tumores de próstata ^49^^,^^50^, cerebro ^51^^-^^53^, ovario ^54^^,^^55^, vejiga ^56^^,^^57^, cuello uterino ^58^, páncreas ^59^^,^^60^ y hepático ^61^, entre otros. Numerosos ensayos clínicos de fase I y II han probado la seguridad y eficacia de esta terapia en humanos ^43^^,^^44^. Diversos estudios demostraron que la transducción del gen *HSV-TK* es más segura y efectiva con vectores adenovirales de replicación deficiente en comparación con los vectores retrovirales ^51^^,^^62^.

### 3.3 Respuesta inmune inducida por el sistema AdV-TK/GCV.

Se ha evidenciado que la terapia con HSV-TK/GCV induce inmunidad anti-tumoral ^63^^,^^64^ e incluso, regresión de tumores cerebrales en ratas inmunocompetentes y de neuroblastoma en un modelo murino ^65^^,^^66^. En los tumores tratados se observa una marcada infiltración de células inflamatorias, con predominio de CD4+ y CD8+, lo que sugiere que el sistema HSV-TK/GCV estimula la respuesta inmune antitumoral ^50^^,^^60^. También se observa la sobreexpresión de las moléculas coestimuladoras B7.1 y B7.2, moléculas de adhesión intracelular (ICAM) y moléculas del MHC en los tumores, y la atracción y activación de APCs ^67^^,^^68^.

Durante el tratamiento de tumores con HSV-TK/GCV, se incrementan las citocinas que estimulan a las APCs y a las células T, como IL-2, IL-12, IFNγ, TNFα y GM-CSF, mientras que las citocinas inhibitorias IL-4, IL-6 e IL-10 no se estimulan ^67^. El incremento en la respuesta inmune ha sido demostrado por niveles más altos de células CD8 circulantes activas y por la elevación de IL-12 en suero ^69^^,^^70^, un mediador clave en la respuesta inmune celular contra infecciones virales y tumores malignos ^69^^,^^71^. Los niveles de células NK se han relacionado con los niveles de IL-12, debido a que las células NK son uno de los blancos de acción de esta interleucina ^69^^,^^72^.

Adicionalmente, la proteína TK viral funciona como un súper-antígeno, estimulando un microambiente tumoral altamente inmunogénico ^68^. Esta proteína induce la liberación y presentación de TAAs que pueden ser reconocidos por los linfocitos T y generar una respuesta inmune adaptativa que también conduce a la citólisis de células tumorales y reclutamiento de APCs ^68^. Las APCs activadas inducen la proliferación de células T por la secreción de IL-2 e IL-12 en el sitio del tumor. Todos estos eventos son deseables para conseguir un potente efecto anti-tumoral ^73^^,^^74^. Sin embargo, debe considerarse una observación que resulta contradictoria. Un ensayo clínico de terapia neoadyuvate con HSV-TK/GCV para carcinoma pancreático mediada por un vector adenoviral, mostró niveles intratumorales incrementados de PD-L1 en especímenes analizados después de la resección quirúrgica. Este evento puede disminuir la respuesta de las células T efectoras, pero puede revertirse con la co-administración de inhibidores de PD-1/PD-L1 ^60^. 

Los ensayos clínicos en los que se ha probado la terapia con HSV-TK/GCV mediada por vectores virales de manera única o en combinación con agentes quimioterapéuticos o radioterapia para cáncer de próstata, carcinoma hepatocelular, o glioblastoma multiforme han mostrado progresos sólidos hasta fase III 67,68,75.

### 3.4 Efectos adversos

A pesar de los resultados prometedores, existen algunas desventajas. Los vectores adenovirales deficientes en replicación disparan una fuerte respuesta inmune humoral y celular que limita su duración a un periodo de entre dos o tres semanas. A pesar de esto, estos vectores pueden resultar útiles para aplicaciones terapéuticas en los que un nivel muy alto de expresión transitoria del gen terapéutico es deseable, como ocurriría en la terapia génica del cáncer ^76^. Por otro lado, la expresión de la proteína TK no es específica de tumor. Una opción interesante es el uso de adenovirus que prefieran su replicación en células tumorales usando un promotor específico ^75^. 

En estudios en fase I y II se han mostrado algunos efectos secundarios, como fiebre leve, neutropenia, dolor de cabeza, trombocitopenia, alteración de las enzimas hepáticas, entre otros. Afortunadamente, estos eventos son transitorios y fáciles de tolerar ^49^^,^^51^^,^^62^.

## 4. Combinación de terapias como nuevo tratamiento

El tratamiento combinado de terapia génica e inmunoterapia es una opción atractiva posibilitada por los avances recientes en la terapéutica del cáncer. El empleo de un sistema de terapia génica suicida determinaría la presentación súbita y masiva de TAAs por un periodo sostenido de semanas o meses. Resulta razonable pensar que esta terapia pueda ser reforzada de manera sinérgica mediante su combinación con un fármaco inhibidor de puntos de control inmunológico administrado de manera sistémica como los descritos en esta revisión. Para ejemplificar más esta idea, se podría suponer que un tumor tratado con HSV-TK/GCV generara la exposición súbita y masiva de TAAs al sistema inmune, los cuales en otras condiciones no generan una inmunoreactividad efectiva por la disminución de MCH I y de moléculas de coestimulación y por la inducción de Treg. Este evento desencadenaría la atracción y activación de las APC y se podría esperar que algunos TAAs induzcan un incremento en el número de linfocitos Treg intratumorales (incluso antes de la intervención con terapia génica), propiciándose un ambiente inmunosupresor. Para prevenir este fenómeno inmunológico y reforzar la respuesta antitumoral, la subsiguiente administración de un inhibidor de punto de control inmunológico, por ejemplo un anticuerpo anti-PD1, afectaría la actividad de las células Treg intratumorales, lo que conduciría a un incremento en la proliferación de las células T efectoras capaces de combatir al tumor y reforzaría una respuesta de memoria inmunológica que potencialmente tendría un efecto protector de largo plazo ^36^. 

Recientemente se ensayó un vector adenoviral portando el gen *HSV-TK* y la secuencia del dominio extracelular de PD-1 fusionada a la porción Fc de la IgG2a de ratón, para producir el segmento PD-1 soluble (sPD1-Ig), que inhibe la actividad del ligando completo de manera competitiva, y por consecuencia, inhibe el efecto apoptótico de las células T mediado por la interacción inmunosupresora del ligando íntegro con su receptor. Éste se administró en un modelo murino de carcinoma de colon y demostró una sinergia entre la terapia HSV/TK y el bloqueo competitivo de la unión PD1/PD-L1. Hubo un decremento significativo en el volumen tumoral en el grupo de ratones tratados con HSV-TK/sPD1 en comparación con los grupos controles, incluyendo el grupo de ratones tratados con el esquema simple HSV-TK/GCV ^77^. 

Es posible que en un futuro cercano se continúen realizando ensayos preclínicos y clínicos que prueben hipótesis similares a la que se plantea en este apartado y que seguramente tendrán resultados clínicos muy efectivos, y sobre todo, con un alto nivel de selectividad terapéutica, lo cual favorecerá aún más el desarrollo de la medicina de precisión en el área de la oncología.

## Conclusión

La potencialización de la respuesta inmune contra los tumores podría ser una estrategia clave para combatir el cáncer. El sistema TK/GCV induce la presentación masiva de TAAs de manera efectiva. Por otra parte, la expresión del súper-antígeno TK facilita la inmunidad celular antitumoral. El uso de anticuerpos monoclonales contra puntos de control de la respuesta inmune, como CTLA-4 y PD-1, contrarrestan la inmunosupresión ocasionada por el tumor. Hasta ahora ambas estrategias se encuentran en ensayos clínicos y han demostrado resultados prometedores. Se esperaría que la combinación de estos dos tipos de terapias fuera sinérgica, más selectiva y efectiva y que tuviera un efecto protector de largo plazo.
